# Changing What You See by Changing What You Know: The Role of Attention

**DOI:** 10.3389/fpsyg.2017.00553

**Published:** 2017-05-01

**Authors:** Gary Lupyan

**Affiliations:** Department of Psychology, University of Wisconsin–Madison, MadisonWI, USA

**Keywords:** perception, attention, top–down processing, knowledge, bistable perception, ambiguous figures, cognitive penetrability

## Abstract

Attending is a cognitive process that incorporates a person’s knowledge, goals, and expectations. What we perceive when we attend to one thing is different from what we perceive when we attend to something else. Yet, it is often argued that attentional effects do not count as evidence that perception is influenced by cognition. I investigate two arguments often given to justify excluding attention. The first is arguing that attention is a post-perceptual process reflecting selection between fully constructed perceptual representations. The second is arguing that attention as a pre-perceptual process that simply changes the input to encapsulated perceptual systems. Both of these arguments are highly problematic. Although some attentional effects can indeed be construed as post-perceptual, others operate by changing perceptual content across the entire visual hierarchy. Although there is a natural analogy between spatial attention and a change of input, the analogy falls apart when we consider other forms of attention. After dispelling these arguments, I make a case for thinking of attention not as a confound, but as one of the mechanisms by which cognitive states affect perception by going through cases in which the same or similar visual inputs are perceived differently depending on the observer’s cognitive state, and instances where cuing an observer using language affects what one sees. Lastly, I provide two compelling counter-examples to the critique that although cognitive influences on perception can be demonstrated in the laboratory, it is impossible to really experience them for oneself in a phenomenologically compelling way. Taken together, the current evidence strongly supports the thesis that what we know routinely influences what we see, that the same sensory input can be perceived differently depending on the current cognitive state of the viewer, and that phenomenologically salient demonstrations are possible if certain conditions are met.

## Introduction

The debate over whether cognition affects perception is in full swing (Stokes, 2013; Lupyan, 2015a; Raftopoulos, 2015a; Zeimbekis and Raftopoulos, 2015; Firestone and Scholl, 2016; Ogilvie and Carruthers, 2016; Teufel and Nanay, 2016). Is what we perceive influenced by our current goals, knowledge, and expectations (e.g., Hohwy, 2013; Goldstone et al., 2015; Lupyan, 2015a; Teufel and Nanay, 2016)? Or is perception composed of encapsulated systems, following their own laws and logic, independent of what the perceiver knows and their current cognitive state (e.g., Pylyshyn, 1999; Orlandi, 2014; Firestone and Scholl, 2016)? The debate spans a variety of issues from how to distinguish cognition from perception to what counts as knowledge to whether the empirical target should be about objective behavior on perceptual tasks or subjective perceptual appearance. All are important questions. The present paper focuses on two aspects of the debate. First, should attentional effects on perception count as instances of cognitive penetrability of perception (CPP)? Second, what is the connection between effects of attention on perception to effects of various kinds of cues on perception? Is cuing perception “just” cuing attention? And if so, what does it tell us about CPP?

The crux is this: The same sensory input or set of inputs can produce different perceptual experiences depending on the attentional state of the viewer. Since attention is a cognitive process (see Is Attention Really Cognitive?), attentional effects ought to constitute *prima facie* evidence that perception is cognitively penetrable. Yet many have argued that demonstrating that cognition *really* influences perception needs to exclude the possibility of the effect being merely attentional (e.g., Pylyshyn, 1999; Macpherson, 2012; Deroy, 2013; Raftopoulos, 2015a; Firestone and Scholl, 2016). After describing the background and rationale of this argument, I try to make explicit some of the assumptions on which it rests, and argue that these assumptions are contradicted by what we know about how attention works. I then go through a number of demonstrations of how the same sensory inputs can be perceived in different ways and discuss the relationships between effects of attention, effects of background knowledge, and effects of cues on perception.

### Is Attention Really Cognitive?

Perhaps the most obvious reason for thinking that attention, that is, the process of attending, is a cognitive process is that when presented with some sensory input it is to possible to volitionally choose what we attend. It is also possible to instruct someone to attend to one thing versus another with immediate consequences for what the viewer ends up seeing (Mack and Rock, 1998; Ward and Scholl, 2015). Just as with many aspects of our cognition, attention is not under complete volitional control. Certain salient sensory events such as a sudden appearance of an object may cause people to automatically attend to the event whether they want to or not (Theeuwes, 2004; Theeuwes et al., 2004). Relatedly, attending to the same salient target has been shown to become easier when it is repeated (priming of pop-out)—a process at one time thought to be similarly automatic and not penetrable to an observers expectations or goals (Maljkovic and Nakayama, 1994).^1^

Vision scientists once thought that it was possible to produce a set of features that are the targets of attentional mechanisms. In the visual domain, dimensions such as spatial frequency and motion direction do appear to be better targets for attentional selection than more complex attributes (Wolfe and Horowitz, 2004) and can thus be fairly viewed as “basic.” However, attempts to derive a complete set of features that form the targets of attentional selection and which divide pre-attentive perception from post-attentive perception have not been successful (e.g., Wolfe, 1998). Recent work has demonstrated that attention is not limited to any closed set of (ostensibly non-semantic) perceptual features such as a spatial frequency and orientation in the case of vision, but extends to clearly semantic attributes such as our knowledge of letters (Nako et al., 2014a), words (Dell’Acqua et al., 2007), and common objects (e.g., Lupyan, 2008; Lupyan and Spivey, 2010; Nako et al., 2014b). That people can attend to such clearly semantic categories means that attention makes use of learned object knowledge making it impossible to reduce attention to a process of selection of basic non-semantic features (see also Goldstone and Barsalou, 1998; Schyns et al., 1998).

### Does Attention Really Affect What We See?

Attending to different things has far-reaching effects on perception. At its most basic, cuing someone to attend to the left makes it easier to see what is on the left (Posner et al., 1980). Such spatial attention is often the sole focus in discussions of attention and CPP (Macpherson, 2012; Deroy, 2013), but it is also possible to attend to *features* in parallel across the visual field with the effect of improved ability to locate task-relevant stimuli (Maunsell and Treue, 2006), and, as further discussed in Section “Cuing Perception: Attention as a Mechanism by Which Knowledge Affects Perception”, to attend to semantic categories (Lupyan, 2008; Çukur et al., 2013; Nako et al., 2014a; Boutonnet and Lupyan, 2015)

Attending not only improves objective performance, but in some cases demonstrably changes subjective perception, enhancing contrast (Carrasco et al., 2004), saturation (Fuller and Carrasco, 2006), and changing perceived size of attended stimuli (Gobell and Carrasco, 2005). Failing to attend to something in the right way can make the difference between seeing and not seeing (hence the term ‘inattentional blindness’) (Mack and Rock, 1998; Ward and Scholl, 2015).

Attentional influences are observed “early” in both place within the visual hierarchy, and time, arguably precluding the existence of truly pre-attentive perception (Foxe and Simpson, 2002; Reynolds and Chelazzi, 2004; Hayden and Gallant, 2005). Although once controversial, it is now common knowledge that attention permeates perceptual processing through and through: from at least the thalamus in the case of mammalian vision (Reynolds and Chelazzi, 2004; Jack et al., 2006; Silver et al., 2007) and down to the cochlea in the case of audition (Smith et al., 2012). We can now say with certainty that many forms of attention work by altering the response profiles of neurons that respond to sensory inputs thereby altering (at least during certain temporal windows) visual representations (Gandhi et al., 1999; Lamme and Roelfsema, 2000; Corbetta and Shulman, 2002; Ghose and Maunsell, 2002; Maunsell and Treue, 2006; Silver et al., 2007). Although the present paper cannot do justice to the vast literature on the perceptual effects of attention (see Carrasco, 2011 for review), it would not be an exaggeration to say that no part of perceptual processing is immune from attentional effects.

## Why Some Believe Attentional Effects Do Not Count As Evidence of Cognitive Penetrability of Perception

And so, we have the following curious situation: attention, a cognitive process affects perception. What we perceive when we attend to one thing is different from what we perceive when we attend to another thing. Yet, it is frequently argued that attentional effects do not count as cases of cognitive penetrability of perception (Pylyshyn, 1999; Macpherson, 2012; Deroy, 2013; Raftopoulos, 2015b; Firestone and Scholl, 2016). The next two sections describe two main reasons for excluding attentional effects from being considered cases of CPP: attention as something that happens *after* perception, and attention as something that happens *before* perception.

### Attention as a Post-perceptual Process

**FIGURE 1:**
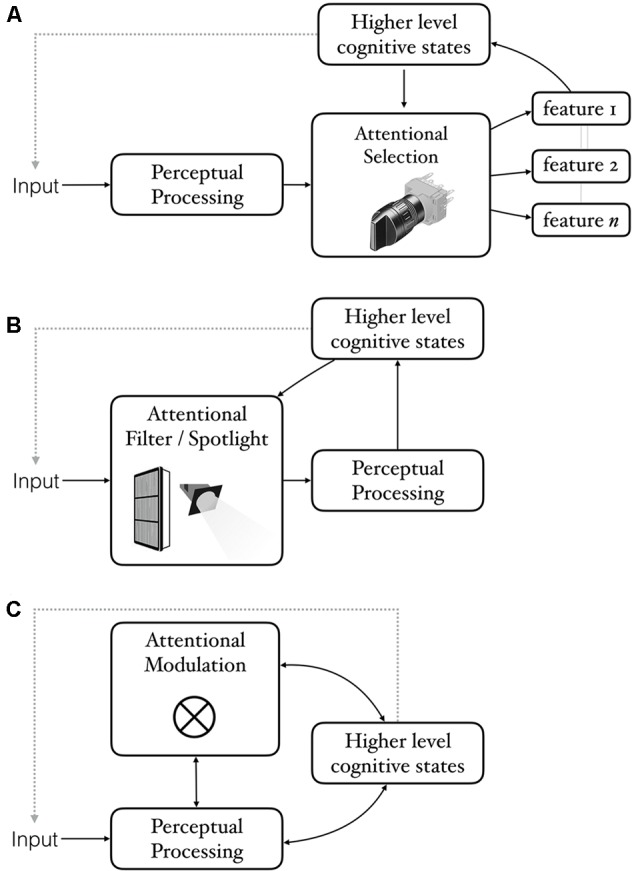
**Three ways of construing the relationship between attention, perception, and cognition.** In all cases, cognitive states can influence what we perceive by literally changing the input for example, via eye-movements. **(A)** Attention as selection that works on the output of a pre-attentive perceptual processing module. Attention construed in this way can be relevant to CPP insofar as perceptual behaviors that one is interested in (e.g., being aware of what one sees) require attention. **(B)** Attention as a pre-perceptual filter or spotlight that shapes input to perception. Attention construed in this way is relevant to CPP insofar as the filters are not limited to content-neutral dimensions such as location, but influence processing in a content-specific (semantically coherent) manner. **(C)** A more general construal of attention as a modulator of perception (symbolized by the symbol for convolution). Some perceptual processes may involve more attentional modulation than others. Cognitive states can influence perception via attention or in other ways. Both routes constitute genuine cases of CPP insofar as the influence is semantically coherent rather than content-neutral.

The first reason for denying that attentional effects counts as evidence of CPP is to view attention as a process of selection happening *after* perceptual processing (often referred to as late-selection; **Figure 1A**). On such a view, perceptual processing may proceed in the same way regardless of what we are attending, with attention determining what contents are selected from perception. For example, Palmer et al. (1993) ask “to what extent attention affects perception rather than memory and decision?” As an illustration of a kind of attention that is well-characterized by post-perceptual selection, imagine someone scanning the walls of an art gallery trying to find the Picassos. To accomplish this, the visual system must process each painting to a sufficient degree so that, at minimum, Picassos can be distinguished from the rest. If one assumes that our knowledge of what Picassos look like resides outside of the visual system, then the best the visual system can do is deliver a ‘percept’ to whatever downstream system has the requisite knowledge. That system can in turn send a signal to examine the painting further, reject it outright as an obvious non-match, and so on. A classic example of a situation often characterized in just such a way is the process of attending to a conversation in a noisy room. Although we may have the impression that we are listening only to the voices of the people we are conversing with, on hearing our name, our focus of attention may suddenly be jerked away to another corner of the room. For this to happen, we must have been processing the ambient speech all along, at least to the level of distinguishing one’s name from all other words. Notably, such recognition of unattended conversation is hardly ubiquitous, happening only about a third of the time, and more so in people with poorer working memory (Conway et al., 2001). More generally, the locus of selection is not fixed, but depends on factors like perceptual and attentional load (e.g., Lavie and Tsal, 1994). Findings like these helped resolve the longstanding debates between early and late-selection (Lavie, 2005).

Still, to the extent that attention sometimes just *selects* stimuli that have already received full perceptual processing—“a subtle form [of] choosing what to perceive” (Macpherson, 2012)—one may conclude that it is therefore of little relevance to questions about effects on perception itself.

### Attention as a Pre-perceptual Process

One reason why many researchers studying perception are so interested in attention involves modulation of perception rather than just a process of selecting amongst fully processed perceptual states. It is possible to be cued (by an experimenter or to cue oneself) to attend to a particular place, feature, or category, with the result that of being objectively *better* at perceiving. Not just remembering, not just better knowing what to do, but *perceiving* better (Carrasco, 2011).

Critics, however, have argued that although such effects clearly count as evidence of attention (a cognitive process) changing what we perceive, they do not count as cases of CPP because attention simply changes the input to the perceptual system. On this view, attention is something that happens *before* perception (**Figure 1B**). The perceptual system then goes on responding to the altered input in a reflexive and modular way encapsulated from the viewer’s knowledge, goals, and expectations. This argument is very clearly expressed by Firestone and Scholl (2016) who argue that attentional effects can be equated to more obvious changes in input like closing or moving one’s eyes:

…there is a trivial sense in which we all can willfully control what we visually experience, by (say) choosing to close our eyes (or turn off the lights) if we wish to experience darkness. Though this is certainly a case of cognition (specifically, of desire and intention) changing perception, this familiar “top-down” effect clearly isn’t revolutionary, insofar as it has no implications for how the mind is organized — and for an obvious reason: closing your eyes (or turning off the lights) changes only the input to perception, without changing perceptual processing itself.

…changing what we see by selectively attending to a different object or feature … seems importantly similar to changing what we see by moving our eyes (or turning the lights off). In both cases, we are changing the input to mechanisms of visual perception, which may then still operate inflexibly given that input.

### Attention as Confound versus Attention as Mechanism

To summarize the argument thus far: there are two broad objections to including attentional effects as instances of CPP. The first objection is that to attending to something involves selecting among already formed perceptual representations (**Figure 1A**). The second objection is that attention simply changes the input to perception. This of course changes what we see, but only because of a difference in input (**Figure 1B**). A related proposal is that attention “rigs up” perception without altering it (Raftopoulos, 2015b).

The first objection—attention is post-perceptual selection—faces two problems. First, although it may indeed be accurate to characterize some attentional effects in this way, it is abundantly clear that much of attention is not simply selection and operates by augmenting perceptual processing itself. Second, regardless of how “late” the attentional effect in question may be occurring and how complete the perceptual processing of unattended stimuli may be, one may wish to nevertheless include such cases as candidates for CPP if they concern behaviors that we wish to count as truly perceptual. For example, even if it could be shown that the unattended gorilla (Simons and Chabris, 1999) is fully processed, its phenomenological invisibility may be relevant if we wish to include being aware of what one sees as part of perception.

To understand why the second objection—attention as a change in input—is compelling to some, and where it goes ultimately wrong, we need to examine some of its underlying assumptions. The objection rests on an analogy between a change in input caused by a change to the sensors, e.g., moving one’s eyes to the left to better see what is on the left, or squinting to blur out some details to see the larger picture, with changes in input caused by endogenous attentional mechanisms. The analogy is at least partially justified for spatial attention. Just as moving our eyes toward a target helps us see it, we have long known that shifting attention *covertly*—without moving one’s eyes—can likewise lead to perceptual improvements (Posner, 1980). Covertly attending to a spatial location enhances spatial resolution, improving performance on tasks that benefit from enhanced spatial resolution (Yeshurun and Carrasco, 1998). Covert attentional shifts are closely correlated with eye movements (e.g., Hart et al., 2013) and share common neural mechanisms. For example, electrical stimulation of the frontal eye fields can evoke both saccadic eye movements to specific locations and attentional shifts.^2^ Such findings that make it sensible—on first glance—to conclude that perceptual changes due to attention are just like those caused by changes to changes to eyegaze. As we shall see, the analogy quickly breaks down when we go beyond spatial attention. The domain of spatial attention, however, allows us to better understand *why* a change in input (whether by moving one’s eyes or moving covert attention) would not constitute CPP. The reason is that the change in perception caused by such a change in input is not content sensitive. Insofar as looking to the left helps us see things on the left solely due to a change in what light now enters the eyes, it will be equally helpful for *everything* that is on the left. This improvement is independent of whether our intention was to look to check for oncoming cars or for pedestrians. In the literature on CPP, this is broadly referred to as a lack of semantic coherence between the cognitive state and the resulting percept (Pylyshyn, 1999; see Lupyan, 2015c; Stokes, 2015 for discussion).

As I will argue below, although some types of attentional shifts may lack semantic coherence, this is not the case for other kinds of attentional effects. It is one thing to find that attending to the left adds visual detail to *anything* on the left. But it is quite another to discover that one can attend to a certain object or object category with the perceptual consequence being changed perception of the *content* that is being attended. Note that even if one argued that the reason that attending to, e.g., cars helps one see cars better is through a change in input to perception, such a change would have to involve a content-specific change and is thus a qualitatively different kind of effect than simply seeing better anything in a particular location. This point is discussed in greater detail below, but first I would like to illustrate how easy it can be to confuse confounds with mechanisms when thinking about CPP.

#### A Mini Case-Study of Confusing Confounds and Mechanisms

In an earlier version of the argument that attentional effects are simple changes in input, Fodor (1988, p. 191) uses the following imagined dialog to draw an analogy between changing one’s percepts by changing where one attends and changing one’s heart rate by doing physical exercise:

a: Heart rate is cognitively penetrable! I can choose the rate at which my heart beats.

b: Remarkable; how do you do it?

a: Well, when I want it to beat faster, I touch my toes a hundred times. And when I want it to beat slower, I take a little nap.

b: Oh.

According to Fodor, it is just as silly to argue that attentional effects count as instances of CPP as it is to argue that changing heart rate through exercise counts as a cognitive effect on heart rate. But *why* does speeding up heart rate by doing some toe touches fail as an argument for heart rate being cognitively penetrable? Because—one assumes—the 100 toe touches would speed up heart-rate to the same extent regardless of whether one’s *intention* was to speed up the heart rate or to stretch one’s hamstrings. There is a lack of semantic coherence. But consider that it is also possible to speed one’s heart rate simply by thinking certain thoughts. No toe touches required (Manuck, 1976; Peira et al., 2013). But suppose that the way one influences heart rate is by thinking about doing exercise. Does this qualify as heart rate being cognitively penetrable? If not, why not? One may argue that it is actually the thoughts about exercise that are causing the heart rate increase rather than the thoughts about increasing one’s heart rate. But this is a strange objection. Perhaps thoughts about exercise are the *mechanism* by which we can cognitively regulate our heart rate.

For argument’s sake, let us assume that thinking about exercise is hacking the heart-rate control system and so does not count as a true cognitive influence. Consider then the following case. Pollo et al. (2003) showed that administering a placebo analgesic reduced the perceived pain of an electric shock to the forehead while also reducing the subject’s heart rate. In other words: when subjects had a placebo-induced belief of being administered a pain-killer, they not only experienced less pain, but a decrease in heart rate. On investigating the mechanism underlying this effect Pollo et al. (2003) discovered that administering an opioid antagonist negated the placebo’s effect on both pain and heart rate, suggesting that the placebo-induced expectation of pain-relief produced a release of endogenous opioids which had the effect of reducing pain and heart-rate, an effect that blocking the opioid receptors could negate.

At this point, a critic may point out that it wasn’t *really* the subject’s cognitive state that reduced their heart rate, but rather the endogenous opioids. But if a person’s beliefs and expectations (which are themselves physical states) are to have an effect on some physiological response, it must happen through some mechanism or another! The endogenous opioids released as a result of the placebo are not a confound. They are part of the mechanism by which placebo analgesics work. It could have turned out that the mechanism is different (and indeed, Pollo et al. describe a different mechanism for placebo effects on ischemic arm pain). And so, it’s the same with attention. To the extent that attention is a key mechanism of how perception performs its function of “providing a description that is useful to the viewer” (Marr, 1982, p. 31), to exclude attentional effects from consideration as cases of CPP is to confuse confounds with mechanisms.

## Perceiving the Same Input in Different Ways: Attentional and Knowledge-Based Influences

In this section, I delve into some details of the interplay between perception, attention, and higher-level cognitive states (**Figure 1C**). My main focus will be on cases of bistable or ambiguous perception as they allow us to keep the physical stimulus the same while changing the observer’s knowledge and/or attentional state. In Section “The Role of Attention and Knowledge in the Perception of Simple Ambiguous Figures”, I discuss some of the ways that attention and prior knowledge influence our perception of bistable images. Some of these may be dismissed as “just” changes in input or post-perceptual selection, but others cannot be. In Section “What Makes Some Perceptual Interpretations Better Than Others?” I sketch in broad strokes a way of thinking about what makes some perceptual interpretations better than others and how attention and knowledge can make a particular interpretation more or less “good.” In Section “Cuing Perception: Attention as a Mechanism by Which Knowledge Affects Perception”, I use the framework developed in Section “What Makes Some Perceptual Interpretations Better Than Others?” to discuss how in-the-moment attentional cues influence what we see, and argue for attention as one of the mechanisms by which knowledge affects perception.

### The Role of Attention and Knowledge in the Perception of Simple Ambiguous Figures

**FIGURE 2:**
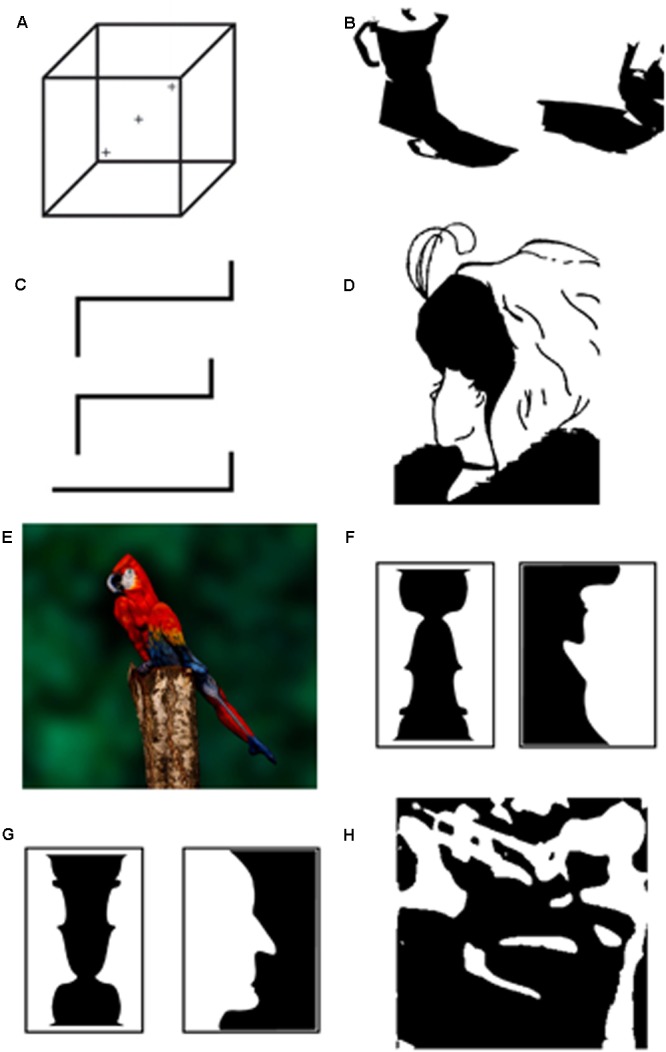
**A variety of bistable and ambiguous images (see text for descriptions of panels **A–H**)**.

If perception is cognitively penetrable, we should be able to find cases where the same physical input can be perceived differently depending on the cognitive state of the perceiver. The existence or ambiguous or bistable images of the kind shown in **Figure 2** provide a natural starting point. That visual bistability is a perceptual phenomenon is supported by both the phenomenological potency of viewing bistable displays and by studies of its neural correlates (e.g., Tong et al., 1998; Meng and Tong, 2004; Kornmeier and Bach, 2005, 2012).

That there are images that can be perceived in multiple ways is not necessarily relevant to the CPP thesis. Consider what is perhaps the best known example of bistability—the Necker cube (**Figure 2A**). The Necker cube can be perceived as extending in depth in two mutually exclusive ways: as if the viewer is looking at it from the top or the bottom. The same 2-dimensional image has two different three-dimensional interpretations indicating that there is a many-to-one projection between 2-dimensional images and 3-dimensional objects. The situation becomes relevant to the CPP thesis if the same 2-dimensional image can evoke a different 3-dimensional interpretation depending on the viewer’s cognitive state.

An effective way of inducing a switch between the two interpretations of the Necker cube is to look at different parts of the image. For example, looking at the right cross in **Figure 2A** causes most viewers to perceive the cube as if looking at it from the top, while looking at the left cross causes most viewers to perceive the alternate perspective. Of course this is utterly unconvincing as a demonstration of CPP. The reason is an apparent lack of semantic coherence. One assumes that the effect of looking at the left or right cross would bias perception in the same way regardless of viewer’s cognitive state.

But it turns out that the *intention* to see the cube in one way or another not only independently affects which 3-dimensional one sees, but has a considerably *larger* effect on the interpretation than where one looks (Hochberg and Peterson, 1987; Toppino, 2003; Meng and Tong, 2004; see also Peterson and Hochberg, 1983; Liebert and Burk, 1985; Peterson, 1986). What about covert attention? In contrast to eye movements, these are more difficult to control. One solution is to present a viewer with a very small Necker cube for which covert or overt attentional shifts ought to be less consequential. Toppino (2003) carried out this experiment and found that the viewer’s intentions had equally large effects when viewing a cube in which the critical areas spanned less than 1° of visual angle compared to a cube an order of magnitude larger.^3^

Recall that what makes Necker cube ambiguous is that the 2-dimensional rendering of the cube is equally compatible with two 3-dimensional interpretations. The fluidity with which we construct 3-dimensional percepts from 2-dimensional inputs makes it tempting to think that although *which* 3-dimensional percept we see at a given time can be influenced by expectations and task-demands, the generation of the percepts themselves is not subject to our knowledge and expectations (Pylyshyn, 1999). But is this true? The very ability to see shapes like the Necker cube as being 3-dimensional is not hardwired. It depends on having had sufficient visual experience (Gregory and Wallace, 1963). For example, one individual who regained sight after being blind between the ages of 3 and 43 described the Necker cube as a “square with lines” (Fine et al., 2003). Still, it may be argued that given sufficient visual experience, the visual system matures sufficiently to allow the process of computing depth from 2-dimensional cues to function in an automatic bottom-up way free of further influences of knowledge and expectations. But this is not so. Both images in **Figure 2B** are 2-dimensional and composed of all the same elements. To the “early” visual system the two objects should look much the same. Yet, the left object is readily seen as a 3-dimensional espresso maker casting a shadow while the object on the right continues to look 2-dimensional (Moore and Cavanagh, 1998). The availability of the 3-dimensional interpretation that is competing (and in this case, quickly winning) when viewing the espresso-maker is simply unavailable for the object on the right until one gains appropriate experience such as glimpsing an enriched grayscale depiction that makes its 3-dimensionality easier to see (see also Sinha and Poggio, 1996).

**Figure 2C** shows another example of an ambiguous image of striking simplicity. When shown this image, the majority (28/50) of participants recruited from Amazon Mechanical Turk reported seeing a 2-dimensional figure (Lupyan, unpublished data). Of these, 61% described it in terms of lines and angles and 39% described it in terms of higher level units such as a staircase or sideways alphanumeric characters: an L and two Zs, or two Zs and a 7. But there is a 3-dimensional alternative that was apparent to the remaining 22/50 observers: an embossed letter E. It is possible, of course, that the ability of the latter group to perceive the alternate interpretation is strictly due to differences in perceptual experiences. Perhaps people who see the embossed letter are those who have previously seen many more embossed letters and therefore are better at recognizing them. But when a separate group of 50 participants were presented with the very same image and informed that it was possible to see it as a letter, about 92% were able to see the embossed E, showing that—controlling for prior perceptual experience—simple verbal cues can affect what people see.

**Figure 2D** shows a different kind of ambiguity. Here, the bistability is between two meanings that can be constructed from the same visual input by assigning the same contours and feature to different parts: the chin in one alternative is the nose in the other. Switching between the two interpretations can be aided by selectively attending to different parts of the image, but can also be accomplished by nonvisual cues, e.g., hearing a voice of a young woman prior to seeing the display (Hsiao et al., 2012). **Figure 2E** poses a similar problem to **Figure 2D** except that seeing the alternative to the initially dominant parrot requires a more significant restructuring of the scene. The alternatives are now not between two kinds of faces, but between a typical-looking parrot and a very atypical woman in body paint. After accomplishing this restructuring, the viewer now has a second stable interpretation that can begin to compete with the initial interpretation (Scocchia et al., 2014). An interesting and to my knowledge untested possibility is that it is only after this initial restructuring that the second interpretation become a target for effective attentional selection.

Another example of a basic visual process being affected by knowledge is shown in **Figures 2F,G**. Lest our visual system be limited to processing a small number of fixed inputs, it is critical to have a way of parsing inputs into constituent (and generative) parts. The most basic way to parse a visual input is by distinguishing the from the ground. How can we tell what is the figure and what is the ground in **Figure 2F**? The solution originally conceived by the Gestalt psychologists is to formulate a set of perceptual ‘laws’ (or biases) such as: objects occupy less area than the ground, objects are generally enclosed and form contiguous regions, objects often have symmetrical contours. Notice that none of these make any mention of object *meaning* and do not take into account prior experience with the candidates for object-hood. As predicted by these Gestalt grouping principles, in **Figure 2F**-left it is easier to see the center black region as the figure than to see the white “surround” as the figure. In **Figure 2F**-right, the situation is more ambiguous; the black and white regions appear to make equally good figures. But consider what happens when the figures are rotated by 180° (**Figure 2G**). The Gestalt dispreferred regions now appear as figure in **Figure 2G**-left, while in **Figure 2G**-right, the white and black regions are now unambiguously perceived as figure and ground, respectively (Peterson et al., 1991). This basic finding and the subsequent work by Peterson and colleagues (Peterson and Gibson, 1994; Trujillo et al., 2010; Cacciamani et al., 2014) provides an obvious challenge to explaining figure-ground segregation using perceptual laws that are not sensitive to content. The relevance of such findings to CPP is that they show that figure-ground segregation does *not* operate in a content-neutral way and is sensitive to at least some aspects of *meaning* (see Peterson, 1994; Vecera and O’Reilly, 1998 for discussion). ^4^ Results such as these also challenge accounts on which perception proceeds through a series of serial operations with earlier ones informationally encapsulated from the results of later ones. Indeed, the idea that object knowledge affects figure-ground segregation appear downright paradoxical if one assumes that the process of figure-ground segregation is what provides the input to later object recognition processes (see also Lupyan and Spivey, 2008; Kahan and Enns, 2014). But finding that recognition can precede and influence such “earlier” perceptual processes is exactly what one would expect if the goal of vision to provide the viewer with a useful representation of the input (Marr, 1982), and to do so as quickly as possible (Bullier, 1999).

**Figure 2H** provides another example of the role that prior knowledge can play in constructing meaning from an otherwise meaningless visual input. Often called “Mooney images” (Mooney, 1957) such two-tone images can be *seen* perfectly well, but the majority of people, most of the time, are unable to perceive anything of meaning in the image.^5^ In the case of **Figure 2H**, approximately 10% of viewers spontaneously perceive the meaningful object. The situation changes dramatically when people are provided with a verbal hint. Told that there is a musical instrument in the image, about 40% quickly see the trumpet. Such verbal cues not only improve recognition, but have additional perceptual consequences. Perceiving the image as meaningful helps people perform a simple perceptual task—determining whether two Mooney images are identical or not. These behavioral improvements were related to differences in early visual processing (specifically, larger amplitudes of the P1 EEG signal, Samaha et al., 2016; see also Abdel Rahman and Sommer, 2008). Contra Pylyshyn’s (1999, p. 357) statement that “verbal hints [have] little effect on recognizing fragmented figures”, we find that not only do verbal hints greatly enhance recognition, but they facilitate visual discrimination.

### What Makes Some Perceptual Interpretations Better Than Others?

Despite the important differences between the cases shown in **Figures 2A–H**, there is something to be gained by attempting to unify them through the lens of perception as an inferential process—a process of generating and testing hypotheses (Gregory, 1970; Barlow, 1990; Rao and Ballard, 1999; von Helmholtz, 2005; Enns and Lleras, 2008; see also Clark, 2013; Hohwy, 2013 for overviews). For example, at the level of object representations, the Necker Cube generates three hypotheses: (a) a 2-dimensional collection of lines, (b) 3-dimensional cube extending up, and (c) a 3-dimensional cube extending down. Hypothesis (a) is dispreferred because it leaves too much unexplained. Accepting (a) would mean that the angles and lines are arbitrary. Hypotheses (b) and (c) offer a simpler description: what explains the arrangement of the lines is that they correspond to a cube. These two hypotheses are equally good at accounting for the arrangement of the lines, but yield mutually exclusive percepts and as a result begin to oscillate (see Hohwy, 2013 for general discussion; see Rumelhart et al., 1986; Haken, 1995; Sundareswara and Schrater, 2008 for examples of computational models).

In the language of predictive-coding, for someone with normal viewing history, hypothesis (a) has higher surprisal (lower ‘goodness’) than hypotheses (b) or (c). In **Figures 2F,G**, a segregating the figure from the ground should take object semantics into account because semantics affects the likelihood that a given feature corresponds to an actual object. We can apply the same principles of predictive coding to better understand what is happening in **Figures 2C,H**. Representing these as a meaningless collection of arbitrary lines results in a less compressible representation than representing them as meaningful objects (see Pickering and Clark, 2014 for a discussion of the relationship between predictive coding and compressibility). This attempt to ‘explain away’ sensory inputs in as compact way as possible is a common foundation of the various predictive-coding models of perception (van der Helm, 2000; Huang and Rao, 2011; Friston et al., 2012), with preference for simplicity going well beyond perception (Chater and Vitanyi, 2003; Feldman, 2003).

In attempting to ‘explain away’ **Figures 2C,H**, however, a hypothesis corresponding to meaningful objects is simply unavailable to most people. As soon as one becomes available, e.g., as a result of a verbal hint, the hypotheses dominates perception and we see the previously meaningless collection of lines as something meaningful percept (an embossed E, a trumpet) (see Christiansen and Chater, 2015 for a discussion of this same idea of continuous re-coding of input into chunks in the domain of language processing).

Although beyond the scope of this paper, it is worth noting that Gestalt principles and other “laws of perception” are not in conflict with theories focusing on minimization of prediction error. The latter theories can be seen as attempting to explain perceptual laws in more general terms. For example, a perceptual “law” such as common fate (wherein separate features all moving together against a background are likely to be grouped into a single object) can be thought as minimizing surprisal/ prediction error by positing a hypothesis that the moving parts can be predicted by a single cause—their belonging to one object. This hypothesis is preferable to the more complex alternative (corresponding to higher surprisal/prediction error) of there being multiple independent causes to the common motion. Our resulting percept of a single moving object is the phenomenological consequence of that simpler hypothesis being preferred.

Learning to associate certain visual inputs with meaningful categories: faces, letters, espresso makers, body-painted women, trumpets, etc., makes these richer hypotheses available as potential alternatives. We (our visual system) can evaluate the likelihood that an input corresponds not just to a visual object, but to a trumpet, or the letter E. These alternatives are preferred to the extent that they offer stronger predictive power, explaining for example, the observed placement of the various visual features. Allowing vision to benefit from these higher-level hypotheses helps make meaning out of noise.

### Cuing Perception: Attention as a Mechanism by Which Knowledge Affects Perception

In discussing how visual knowledge can lead observers to perceive the same sensory input in different ways, I conflated two kinds of effects of cognition on perception. The first concerns the finding that previous experiences with letters, faces, and various objects look like can influence the operation of even basic perceptual processes like figure-ground segregation and construction of 3-dimensional structure. The second is that it is sometimes possible to change what one sees through various cues. For example, the likelihood that people perceive **Figure 2C** as a single three-dimensional object is affected by being told that it is possible to see it as a letter. Some critics of CPP contest that CPP of the first type (sometimes called “diachronic penetrability”, see McCauley and Henrich, 2006 for discussion) is not *really* evidence of CPP because it merely shows that such visual knowledge has become incorporated into the visual system over time at which point it (apparently) no longer counts as cognitive. I will forego discussing this rather odd argument. Instead, in this section, I elaborate on the second kind of effect—sometimes called synchronic penetrability—wherein similar or even identical inputs are perceived in different ways depending on the cognitive state of the viewer at the time the input is perceived (see also Klink et al., 2012).

**FIGURE 3:**
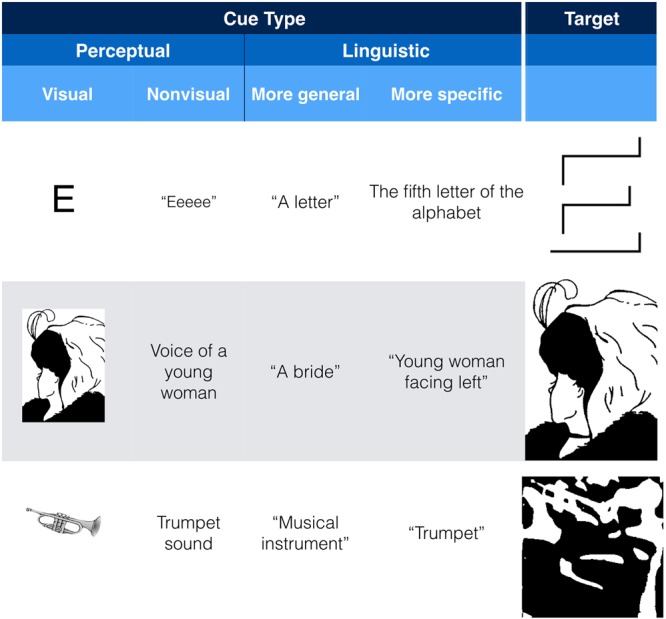
**Examples of perceptual and linguistic cues that can change how the target image is perceived**.

One way to change perception is by using a perceptual cue. For example, to help people see the embossed E in **Figure 2C**, one can cue them with a conventional letter “E” (**Figure 3**, top row). To bias people to see the young woman in **Figure 2D**, one can cue them with a biased version of the figure (**Figure 3**, middle row), and to help people see the trumpet in the Mooney image in **Figure 2H**, one can cue them with a more conventional picture of a trumpet (**Figure 3**, bottom row) or else trace out the outline of the trumpet in the original image.^6^ If such perceptual cues were the only way to affect how an ambiguous or under-determined image is perceived, such cueing effects would be of little relevance to CPP. But there are other ways of cueing perception. For example, I suspect that simply hearing “Eeee” immediately prior to or during seeing **Figure 2C** would increase the likelihood of perceiving it as a single three-dimensional letter E. Similarly, an auditory cue—the voice of a younger or older woman biased people to perceive the younger or older woman in **Figure 2D**, respectively, an effect that was additive with effects of spatial attention (Hsiao et al., 2012). Finally, although not empirically tested to my knowledge, it is conceivable that hearing a trumpet sound can help people see the trumpet in **Figure 2H**.

Such cross-modal effects are sometimes excluded from counting as instance of CPP because, it is argued, they merely show automatic influences of one perceptual modality on another—an intraperceptual effect (e.g., Pylyshyn, 1999, sect. 7.1) rather than an effects of cognition on perception. Drawing such an intraperceptual boundary strikes me as self-defeating, for it would mean that the knowledge as what an “E” sounds like, male and female voices, and musical instruments would all become part of the perceptual system. Someone holding the view that audiovisual integration does not count as CPP may point to the findings of Alsius and Munhall (2013) which show that audiovisual integration can occur in the absence of conscious awareness of the visual stimulus (see also Faivre et al., 2014) as evidence that such integration occurs in a completely automatic way. But this automaticity is not inevitable: even conventional audiovisual integration can be interfered with by having participants engage in an attentionally demanding task (Alsius et al., 2005).

We need not restrict ourselves to literal perceptual cues. The next two columns of **Figure 3** show examples of general and more specific linguistic cues to perception. As mentioned above, being informed that it was possible to see a letter in **Figure 2C** more than doubled the likelihood of people seeing the embossed E, a result that one can speculate would only increase if one was given more precise information via language of what the letter was. In the old-woman/young-woman case, although many people quickly see the ambiguity, the possibility of biasing naïve viewers to one interpretation or another purely through language (i.e., without any overt perceptual cues), and that linguistic instructions continue to be effective in biasing one’s perception (e.g., Hsiao et al., 2012), speaks to the power of language to guide perception in the absence of any overt perceptual cues. In case of the Mooney image depicted in the last row of **Figure 3**, even superordinate linguistic cues like “animal” and “musical instrument” aid in recognition of the images. More specific cues (e.g., the word “trumpet”) are predictably more effective (Samaha et al., 2016). In other work, we have shown using hearing a verbal cue affects visual processing within 100 ms. of visual onset (Boutonnet and Lupyan, 2015), results that we interpret as showing that verbal cues activate visual representations, establishing “priors” that change how subsequent stimuli are processed (Edmiston and Lupyan, 2015, 2017; Lupyan and Clark, 2015).

Here, one may again ask whether the power of language to guide and bias perception is due to changing the input to perception *via* attention. The answer is that it depends on the cue. A location cue like “LEFT” is highly effective in changing where someone attends (Hommel et al., 2001), but because its effect is (presumably) content neutral, it is possible to think of it as merely a change in input. Other linguistic cues, however, have much richer semantic content: hearing “dog” helps people perceive dogs (Lupyan and Ward, 2013; Boutonnet and Lupyan, 2015). One may argue that such effects simply show that language is a good way to “rig up” perception (Raftopoulos, 2015b). But rather than being an alternative to CPP, such an argument speaks to the *mechanism* by which language has its effects. The fact of the matter remains that a person presented with the same sensory input can perceive it in different ways depending on a word they had previously heard (see Lupyan, 2015a for review).

## The “Wow” Factor: When Can We Really See Our Knowledge Impacting Perception?

The evidence for the various ways in which knowledge affects perception keeps growing. Here is a brief sampling: knowledge of how arms and legs are attached to torsos affects perceived depth from binocular disparity information (Bulthoff et al., 1998). Knowledge that bricks are harder than cheese affects amodal completion (Vrins et al., 2009). Recovery of depth from 2-dimensional images depends in part on object recognition (Moore and Cavanagh, 1998, **Figure 2B**) as is the arguably more basic process of figure-ground segregation (Peterson, 1994 for review, see also **Figures 2F,G**). Scene knowledge affects perception of edge orientations (Neri, 2014). Knowledge of the real-world size of, e.g., a basketball affects apparent speed of motion (by altering perception of distance) (Martín et al., 2015). Knowledge of usual object colors shades our color perception (Hansen et al., 2006; Olkkonen et al., 2008; Witzel et al., 2011; Kimura et al., 2013; Witzel, 2016) and influences the vividness of color afterimages (Lupyan, 2015b). Meaningfulness of printed words affects their perceived sharpness and influences our ability to detect changes in sharpness (Lupyan, 2017b). Hearing the right word, can make visible something that is otherwise invisible (Lupyan and Ward, 2013).

In spite of this evidence and the cases described in Section “The Role of Attention and Knowledge in the Perception of Simple Ambiguous Figures”, some critics of CPP remain unmoved. One reason for the continued resistance is that many of these results lack the “wow” factor common to many well-known illusions designed to demonstrate the workings of the visual system. For example, Firestone and Scholl (2015, 2016) ask why, if what we know changes what we see, is it so hard to find cases where one can really *see* these effects for oneself. As a comparison of what it means to see a visual effect for oneself, consider our perception of how bright something is. Naively, one might suppose that it depends simply on the amount of light reflected by a surface (i.e., it’s luminance). That this is not so can be plainly seen in an illusion like the Adelson (1993) Checkerboard in which two surfaces with the same luminance are perceived to have very different brightness.^7^

In this last section I will attempt to explain why demonstrations of CPP tend to be less compelling than conventional visual illusions.^8^ I then provide a recipe for creating phenomenologically compelling demonstrations of CPP and show two examples.

What makes Adelson’s Checkerboard so compelling as an illusion is that it is possible to prove to the observer that it is indeed an illusion by making the perceived difference in lightness to vanish right before the person’s eyes by, for example, joining the two patches or masking the context thus allowing observers to see that their perception of one patch as being much lighter than the other was being produced by factors other than their luminance. Compared to this level of control we have over factors that induce such illusions, our ability to control the cognitive state of the viewer is far more limited. For example, consider the finding that an objectively achromatic picture of a banana looks yellower than a meaningless color patch (Hansen et al., 2006). If perceived color is truly influenced by knowledge of the object’s canonical color (i.e., reflects our memory of previous experiences with the object), then turning off one’s knowledge that one is looking at a banana should affect perceived color. That would make for a compelling demonstration! But it’s not possible to turn knowledge on and off in this way. So what can we do instead?

One solution is to manipulate the strength of the association between the input stimulus and stored representations.^9^ In **Figure 2B**, people readily construct a 3-dimensional representation of a 2-dimensional image when it corresponds to a recognizable object, but not when its low-level features are rearranged into a novel image (Moore and Cavanagh, 1998). The difference in 3-dimensional structure is apparent, but the two stimuli are too different from one another to allow for easy comparison. This has the effect of reducing the “wow” factor because to the viewer it just appears that one of the stimuli is 3-dimensional and the other is not. It does not feel like the difference is caused by one’s knowledge. A method that further minimizes physical changes to the sensory inputs while attempting to manipulate knowledge is simple image rotation (e.g., **Figures 2F,G**, Peterson, 1994). Turning an object upside down maintains all of its low-level visual properties, but weakens its association with a stored higher-level representation (assuming that the object or scene is typically encountered in a canonical orientation). Another way to manipulate knowledge is through cuing. For example, cuing people with an object’s name can enhance the contribution of prior knowledge on perception (Lupyan, 2012; Boutonnet and Lupyan, 2015). A cue can help bias one interpretation over another of ambiguous objects of the kind shown in **Figure 2**. In instances like **Figures 2C,E,H**, it can even introduce new interpretations. But almost by definition, such ambiguous objects tend to be lousy examples of the cued categories. Although our phenomenology of **Figure 2H** is arguably different when we perceive the trumpet, the change is not nearly as phenomenologically compelling as the best visual illusions because the change from a collection of meaningless contours to a collection of contours making up a sketchy outline of a trumpet is too small to elicit a “wow.” The situation is somewhat better in **Figure 2C** because the alternative made accessible by “there is a letter here” cue explains more of the unexplained variation.

**FIGURE 4:**
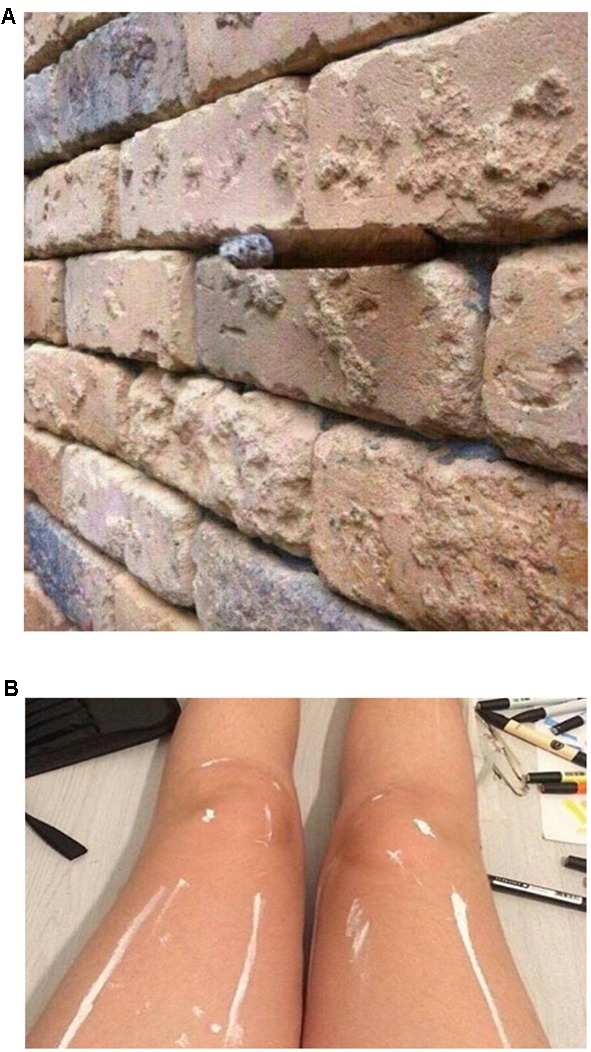
**Two especially phenomenologically compelling examples of how what we know can affect what we see. (A)** A brick wall that, with the right knowledge, can be seen as something else.^10^ **(B)** A pair of legs with two alternative interpretations.^11^ See notes 10 and 11 for hints regarding alternative interpretations of each image.

To maximize the “wow factor” would require a stimulus that is easily seen as one thing and then, provided the right cue, can be seen as a good example of something else. In the language of predictive coding, the initial stimulus ought to yield low surprisal, but following a cue the surprisal should increase causing the visual system to reorganize the image into a new percept with low surprisal. Two such cases are shown in **Figure 4**.

**Figure 4A** (Plait et al., 2016) shows an apparently perfectly normal brick wall. There does not appear to be anything ambiguous or atypical about it. But being informed of an alternative interpretation changes that. The new interpretation (see endnote), makes the original interpretation a poorer fit to the data (i.e., increases its surprisal) while simultaneously making the new interpretation a better fit to the data. And so, on learning of the new interpretation, our percept is altered. I find it next to impossible to now see the image as I initially saw it (interestingly, rotating the image seems to partly disrupt the effect of the newly acquired knowledge).

**Figure 4B** (Krishna, 2016) is another compelling demonstration. Here, people appear to be split on what they initially see (see endnote for the description of the two interpretations). But perhaps because the two interpretations differ considerably in how they account for what is happening with the two legs, and because they both interpretations offer such good, but mutually exclusive accounts of the sensory data, the resulting phenomenological switch when one is cued to the alternative (or discovers it on their own) tends to be more compelling than in the cases of bistability shown in **Figure 2**.

## Summary and Conclusion

Perhaps the simplest way to test the proposition that what we know influences what we see is to find cases where the same sensory input can be perceived in different ways depending on one’s cognitive state, such as what one knows or expects. Findings that attention—a cognitive process—has strong influences on every aspect of perception would seem to provide *prima facie* evidence for cognitive penetrability of perception (CPP). Yet, critics of CPP have discounted attentional effects, arguing that they either reflect post-perceptual selection among fully realized perceptual representations, or pre-perceptual processes that change the input to perception but not perception itself (**Figure 1**). I have argued that although some attentional effects may well be post-perceptual, others are clearly not (Sections “Does Attention Really Affect What We See?” and “Attention as a Post-Perceptual Process”). Some types of spatial attention may indeed be similar to genuine changes in input: attending to the left may be similar to looking to the left in that both improve processing of whatever is on the left regardless of content or the cognitive state that drove the attentional shift. Such attentional effects lack semantic coherence and critics are correct to exclude them from counting as examples of CPP. Other attentional effects, however, do show semantic coherence in that the attentional state is sensitive to content (see The Role of Attention and Knowledge in the Perception of Simple Ambiguous Figures) and so should count as genuine instances of CPP. In Sections “The Role of Attention and Knowledge in the Perception of Simple Ambiguous Figures,” “,” and “Cuing perception: Attention as a mechanism by which knowledge affects perception,” I discussed cases where the same (or similar) visual inputs are perceived differently depending on the observer’s knowledge (**Figure 2**), and the ability to cue knowledge using both perceptual and non-perceptual linguistic cues (**Figure 3**). I then discussed some of the reasons why it is often difficult to experience knowledge and cues affecting perception in a phenomenologically compelling way (see The “wow” Factor: When Can We Really See Our Knowledge Impacting Perception?). Lastly, I provided some arguably compelling examples of being able to see for oneself how knowledge can affect what one sees (**Figure 4**).

Taken together, the evidence licenses several conclusions. First, it is not possible to characterize attentional effects as non-semantic changes in input of the kind that occur when we look at one location versus another. Rather, attention can and often does operate over dimensions that we normally think of as reflecting meaning and these attentional effects should be counted as genuine instances of CPP. Second, the possibility of exogenously cueing one’s knowledge in real time to bias how something is perceived strongly suggests that under normal circumstances what we see is reflecting our endogenous cognitive state. Third, to understand why these effects often lack the “wow factor” common to the best visual illusions, it is useful to work through the effects through the lens of predictive coding. Knowledge ought to change what we see to the extent that it provides a better hypothesis of the sensory data.

## Ethics Statement

The study described in Section “The Role of Attention and Knowledge in the Perception of Simple Ambiguous Figures” was exempted by the University of Wisconsin–Madison Social and Behavioral Science IRB owing to the anonymity of the participants recruited via Amazon Mechanical Turk and the minimal risk posed by the task of reporting their perception of ambiguous figures.

## Author Contributions

The author confirms being the sole contributor of this work and approved it for publication.

## Conflict of Interest Statement

The author declares that the research was conducted in the absence of any commercial or financial relationships that could be construed as a potential conflict of interest.

## Footnotes

1 Subsequent work has shown that even such putatively automatic attentional guidance is modulated by the viewer’s expectations (Leonard and Egeth, 2008; Pascucci et al., 2012) and task relevance of the dimensions to be attended (Wolfe et al., 2003; Fecteau, 2007). More generally, the original formulation of perceptual salience in terms of sensory salience (Itti and Koch, 2000) is being supplanted by formulations that incorporate semantic factors into computatios of salience (e.g., Nyström and Holmqvist, 2008; Wolfe et al., 2011; Wu et al., 2014; Santangelo et al., 2015).

2 Such findings led researchers to formulate the premotor theory of spatial attention on which saccades are covert attentional shifts writ large (see Thompson et al., 2005 for a critical review).

3 Discussing the Necker Cube, Deroy (2013) writes: “Trivially speaking, two persons confronted to the same visual object in the same illumination conditions may not perceive the same thing because they don’t look at it in the same way.” If by “don’t look at it in the same way” Deroy means that people attend to different parts of the image, then the argument does not square with the empirical literature because people *do* in fact see different things even if they attend to the same regions. If by “don’t look at it in the same way” Deroy means that people look at the same image with different expectations and this affects what they see, then that sure sounds like cognition affecting perception.

4 Firestone and Scholl (2015) reinterpret Peterson and Hochberg (1983) finding by arguing that the differences between figure-ground assignment in the familiar and unfamiliar orientations “don’t involve effects of knowledge *per se* [because] inversion eliminates this effect even when subjects know the inverted shape’s identity” (see Section 2.5 of their paper). This argument confuses different senses of knowledge. We may *know* in an intellectual sense that an upside–down outline of a woman is still an outline of a woman, but despite this intellectual knowledge it is still harder to recognize the white shapes as a woman’s silhouette in **Figure 2F** than in **Figure 2G**. The harder the recognition, the less effect the object representation can have on the figure-ground segregation process as it unfolds.

5 One may speculate that people’s difficulty with making sense of such images is analagous to the problem faced by individuals with associative object agnosia when they attempt to make sense of more conventional images (Farah, 1990).

6 That perception can be cued in this way may seem obvious, but we still lack an understanding of how a single perceptual hint can induce a long-term change in the ability to perceive stimuli like the Mooney image in **Figure 2H** as meaningful.

7 The Adelson checkerboard illusion can be viewed at http://web.mit.edu/persci/people/adelson/checkershadow_illusion.html

8 For brevity and to maintain focus on attention, I have avoided discussing the argument that perception is cognitively impenetrable because knowing about an illusion does not (necessarily) make it go away. For discussion, see Lupyan (2015a, Section 5.1) and Lupyan (2017a, Section 6.3).

9 This leads to the prediction that a more realistic banana should activate our color knowledge to a greater degree than a less realistic banana (a point frequently lost in philosophical treatments of CPP that tend to think of knowledge as all or none, e.g., Deroy, 2013). Indeed, the memory color effects are stronger when a viewer is presented with a more realistic grayscale image (Olkkonen et al., 2008; see also Lupyan, 2015b for an effect of weakening associations by turning the image upside-down on the perceived vividness of color afterimages).

10 The “crack” in the wall is not a crack. It is the tip of a cigar which is stuck into the wall, extending outward.

11 Many viewers will see two shiny legs because the combination of visual cues is consistent with an interpretation of skin gloss. But there is an equal or better interpretation: the legs are covered with streaks of white paint.
